# Noncommuting Common Causes, Law of Total Probability, and No-Conspiracy

**DOI:** 10.3390/e28101158

**Published:** 2026-10-21

**Authors:** Gábor Hofer-Szabó

**Affiliations:** ELTE, Research Center for the Humanities, Tóth Kálmán Street 4, 1097 Budapest, Hungary; szabo.gabor@abtk.hu

**Keywords:** noncommuting common causes, law of total probability, no-conspiracy

## Abstract

Noncommuting common causes posited as explanations of quantum correlations violate the law of total probability. On the operationalist interpretation of quantum mechanics, this flaw might be interpreted as a result of the noncommuting common causes being conspiratorial. In this paper, we will show that this is not the case. Noncommuting common causes violate the law of total probability but they are not conspiratorial.

## 1. Introduction

In a classical probability space (Ω,Σ,p) the law of total probability is an identity. The law says that given an event A,a∈Σ and a partition ${\left\{C_{k}\right\}}_{k}$—that is, a set of mutually exclusive events such that ${⋃}_{k}C_{k}=\Omega$—the following equation always holds:

(1) $$\begin{array}{ccc} p(A) & = & \sum_{k}p\left(A|C_{k}\right)\;p\left(C_{k}\right) \end{array}$$

The conditional version of the law of total probability is likewise an identity:

(2) $$\begin{array}{ccc} p(A|a) & = & \sum_{k}p\left(A|a\wedge C_{k}\right),p\left(C_{k}|a\right) \end{array}$$

If, however, we replace the conditional probabilities $p(C_{k}|a)$ in (2) by the corresponding unconditional probabilities $p(C_{k})$, the equation

(3) $$\begin{array}{ccc} p(A|a) & = & \sum_{k}p\left(A|a\wedge C_{k}\right),p\left(C_{k}\right), \end{array}$$

does not hold in general. It does hold if, for all *k*,

(4) $$\begin{array}{ccc} p(a\wedge C_{k}) & = & p\left(a\right)\;p\left(C_{k}\right) \end{array}$$

These simple mathematical facts have important implications for the common causal explanation of quantum correlations. Quantum correlations, just like other correlation between spacelike separated events, call for a common causal explanation [1]. In a series of papers, refs. [2,3,4,5,6] and recently in [7], the role of the so-called noncommuting common causes in the causal explanation of quantum correlations has been explored. This research line was a generalization of the work of [8,9,10] based on an idea of Clifton and [11]. Noncommuting common causes had many virtues but they also had a serious flaw: they violate the law of total probability. This means that the correlating events cannot be reconstructed from the common causes (considered as a screener-off partition in a noncommutative probability space). In other words, noncommuting common causes do not causally explain the correlations.

Now, in the operationalist approach a quantum probability ϕ(A) is interpreted as a classical conditional probability p(A|a) that is the probability of getting a certain measurement outcome provided that a certain measurement is performed. In this reading of quantum probabilities, the violation of the law of total probability simply translates into the impossibility of reconstructing these probabilities from probabilities of outcomes conditioned on the settings *plus* the common causes $p(A|a\wedge C_{k})$. But this is just Equation (3). And as we saw, Equation (3) follows from Equation (4), which also has an important role in causal explanations: it expresses no-conspiracy, the probabilistic independence between the common causes and the measurement choices.

Now, the above connection between (3) and (4) suggests a possible explanation for the violation of the law of total probability in case of noncommuting common causes. It suggests that the reason for the violation of the law of total probability is the violation of no-conspiracy. Noncommuting common causes, interpreted operationally as classical condition probabilities, violate the law of total probability because they are conspiratorial.

The aim of this short paper is to show that this is not the case. Noncommuting common causes indeed violate the law of total probability but they are not conspiratorial. In the paper, we first start from the general noncommutative framework and clarify the connection between noncommuting common causes and the law of total probability (Section 2); then following the operationalist tradition we translate the noncommutative probabilities into classical conditional probabilities and establish the connection between noncommuting common causes and no-conspiracy (Section 3); finally we put together the law of total probability and no-conspiracy (Section 4).

## 2. Noncommuting Common Causes and the Law of Total Probability

A *non-classical probability measure space* is the triple (N,P(N),ϕ) where N is von Neumann algebra, P(N) is the lattice of projections (events) of N, and ϕ is a state on N [12]. Two commuting projections *A* and B∈P(N) are said to be *correlating* in the state ϕ:N→C if

(5) $$\begin{array}{ccc} \phi (AB) & \neq & \phi (A)\;\phi (B). \end{array}$$

A set of mutually orthogonal projections ${\left\{C_{k}\right\}}_{k\in K}\subset P\left(N\right)$ is a *partition of the unit* 1∈N if $\sum_{k}C_{k}=1$. A partition of the unit ${\left\{C_{k}\right\}}_{k\in K}\subset P\left(N\right)$ is called the *common cause* of the correlation (5) if for those k∈K for which $\phi (C_{k})\neq 0$, the following *screening-off condition* holds:

(6) $$\begin{array}{ccc} \frac{\phi (C_{k}ABC_{k})}{\phi (C_{k})} & = & \frac{\phi (C_{k}AC_{k})}{\phi (C_{k})}\frac{\phi (C_{k}BC_{k})}{\phi (C_{k})}. \end{array}$$

If all $C_{k}$ commute with both *A* and *B* for all k∈K, we call ${\left\{C_{k}\right\}}_{k\in K}$ a *commuting* common cause and a *noncommuting* one otherwise.

A common cause can screen off multiple correlations. Let $\left\{\left(A_{m},B_{n}\right);m\in M,n\in N\right\}$ be a set of pairs of commuting projections in P(N) correlating in the sense that

(7) $$\begin{array}{ccc} \phi (A_{m}B_{n}) & \neq & \phi \left(A_{m}\right)\;\phi \left(B_{n}\right). \end{array}$$

A partition of the unit ${\left\{C_{k}\right\}}_{k\in K}\subset P\left(N\right)$ is said to be a *joint common cause* of the correlations (7) if for any k∈K, when $\phi (C_{k})\neq 0$, the screening-off conditions hold:

(8) $$\begin{array}{ccc} \frac{\phi (C_{k}A_{m}B_{n}C_{k})}{\phi (C_{k})} & = & \frac{\phi (C_{k}A_{m}C_{k})}{\phi (C_{k})}\frac{\phi (C_{k}B_{n}C_{k})}{\phi (C_{k})},\;m\in M,n\in N \end{array}$$

Again, if ${\left\{C_{k}\right\}}_{k\in K}$ commutes with $A_{m}$ and $B_{n}$ for all m∈M,n∈N, we call it a *commuting* joint common cause and a *noncommuting* one otherwise.

The difference between commuting and noncommuting joint common causes is particularly clear in the EPR–Bohm scenario which *does not* admit a joint *commuting* common causal explanation but *does* admit a joint *noncommuting* common causal explanation. In the *EPR–Bohm scenario* [13] one prepares pairs of spin-$\frac{1}{2}$ particles in a singlet state and one measures their spin in directions ${\vec{a}}_{m}$ and ${\vec{b}}_{n}$ (m,n=1,2), respectively. The ‘spin-up’ events in both wings are represented in quantum mechanics as projections of the von Neumann algebra of the tensor product matrix algebra $M_{2}\left(C\right)⊗M_{2}\left(C\right)$ with the following probabilities:

(9) $$\begin{array}{ccc} \phi (A_{m}) & = & \langle \Psi |P_{m}^{A}⊗1_{B}|\Psi \rangle =\frac{1}{2} \end{array}$$

(10) $$\begin{array}{ccc} \phi (B_{n}) & = & \langle \Psi |1_{A}⊗P_{n}^{B}|\Psi \rangle =\frac{1}{2} \end{array}$$

(11) $$\begin{array}{ccc} \phi (A_{m}B_{n}) & = & \langle \Psi |P_{m}^{A}⊗P_{n}^{B}|\Psi \rangle =\frac{1}{2}\sin^{2}\left(\frac{\theta_{mn}}{2}\right) \end{array}$$

where $|\Psi \rangle =\frac{1}{\sqrt{2}}\left(|01\rangle -|10\rangle \right)$ is the singlet state vector; $A_{m}=P_{m}^{A}⊗1_{B}$ and $B_{n}=1_{A}⊗P_{n}^{B}$, with $1_{A}$ and $1_{B}$ denoting the units in the two matrix algebras and $P_{m}^{A}$ and $P_{n}^{B}$ denoting the projections onto the eigensubspaces with eigenvalue $+\frac{1}{2}$ of the spin operators $\vec{\sigma }{\vec{a}}_{m}$ and $\vec{\sigma }{\vec{b}}_{n}$ in $M_{2}\left(C\right)$ associated with directions ${\vec{a}}_{m}$ and ${\vec{b}}_{n}$, respectively; $\vec{\sigma }=\left(\sigma_{x},\sigma_{y},\sigma_{z}\right)$; and $\theta_{mn}$ is the angle between ${\vec{a}}_{m}$ and ${\vec{b}}_{n}$. For non-perpendicular directions ${\vec{a}}_{m}$ and ${\vec{b}}_{n}$, (9)–(11) predict correlations which call for a common causal explanation.

But there is no such explanation if the common cause is *commuting*. This is because in this case the four correlating pairs $\left\{\left(A_{m},B_{n}\right);\;m,n=1,2\right\}$ need to satisfy the Clauser–Horne inequality:

(12) $$\begin{array}{c} -1⩽\phi \left(A_{1}B_{1}\right)+\phi \left(A_{1}B_{2}\right)+\phi \left(A_{2}B_{1}\right)-\phi \left(A_{2}B_{2}\right)-\phi \left(A_{1}\right)-\phi \left(B_{1}\right)⩽0, \end{array}$$

(For the proof, see Appendix A.) For certain directions, however, for example for four co-planar directions $\theta_{11}=\theta_{12}=\theta_{21}=45^{\circ }$ and $\theta_{22}=135^{\circ }$, the Clauser–Horne inequality (12) is violated, ruling out a commuting common causal explanation of the correlations $\left\{\left(A_{m},B_{n}\right);\;m,n=1,2\right\}$.

How about *noncommuting* common causes?

First observe that in the last step of the derivation of the Clauser–Horne inequality we used an analogue of the law of total probability

(13) $$\begin{array}{c} \phi \left(X\right)=\sum_{k}\phi \left(C_{k}XC_{k}\right)\;\;X=A_{m},B_{n},A_{m}B_{n} \end{array}$$

which we call *noncommutative law of total probability* [5].

However, the noncommutative law of total probability does not necessarily hold if ${\left\{C_{k}\right\}}_{k\in K}$ is a noncommuting common cause. This means that the violation of the Clauser–Horne inequality does not rule out a noncommuting joint common causal explanation of the correlations between the events $\left\{\left(A_{m},B_{n}\right);\;m,n=1,2\right\}$. And indeed, refs. [4,5] have found a noncommuting common cause for the above four correlations (moreover, a *local* one, that is a common cause localized in the intersection of the causal past of the correlating events). It is important to stress that this common cause was necessarily noncommutative, since the Clauser–Horne inequality excludes the commutative common causes. To avoid the Clauser–Horne inequality, this noncommuting common cause violated the noncommutative law of total probability.

Now, the violation of the noncommutative law of total probability has the following unpleasant consequence which calls into question the very explanation [14,15]. From a philosophical point of view, a common causal explanation of a correlation is nothing but a finer description of the correlation at a more fundamental level. This means that we have two descriptions of the same situation: a fine-grained description including the correlating events and their causes, and a coarse-grained description which includes only the correlating events. And the two descriptions need to be consistent in the sense that the probabilities assigned to the correlating events have to be the same. And this is exactly what is ensured by the law of total probability. If it holds, the probability $\phi (A_{m})$ obtained from the coarse-grained description will be equal to the summed-up probability $\sum_{k}\phi \left(C_{k}A_{m}C_{k}\right)$ obtained from the fine-grained description, similarly for $B_{n}$ and $A_{m}B_{n}$. This means that in the noncommutative case where the noncommutative law of total probability is not an identity, the common causal explanation cannot necessarily be interpreted as a finer description of the situation to be explained.

## 3. Noncommuting Common Causes and No-Conspiracy

The question of the relation of the noncommuting common causes to the law of total probability is a separate question from their relation to no-conspiracy. While noncommuting common causes generally violate the noncommutative law of total probability, they do *not* violate no-conspiracy. To see this, we need to bring in the concept of measurement settings; that is, we need to give an operationalist interpretation of the quantum probabilities.

On the operationalist interpretation of quantum mechanics, the quantum probability ϕ(X) is just a shorthand for a *classical conditional probability of classical events*, that is the probability $p(X_{cl}|x_{cl})$ of getting the measurement outcome $X_{cl}$ provided that a certain measurement $x_{cl}$ is performed. This idea can be made precise by the translation manual called ‘Kolmogorovian Censorship Hypothesis’ [16,17,18].

Let (N,P(N),ϕ) be a non-classical probability space. Let Γ be a countable set of pairwise non-commuting selfadjoint operators in N. Denote by $\left\{X^{Q}\right\}$ the set of spectral projections of *Q*. Finally, let a map $p_{0}:\Gamma \to \left[0,\;1\right]$ be such that

(14) $$\begin{array}{c} \sum_{Q\in \Gamma }p_{0}\left(Q\right)=1,\;\;\;\;\;\;\;\;\;\;\;\;p_{0}\left(Q\right)>0. \end{array}$$

Then, according to the *Kolmogorovian Censorship Hypothesis*, there exists a classical probability space (Ω,Σ,p) such that for every spectral projection $X^{Q}$ of any *Q* there exist events $X_{cl}^{Q}$ and $x_{cl}^{Q}$ in Σ such that

(15) $$\begin{array}{c} X_{cl}^{Q}\subset x_{cl}^{Q} \end{array}$$

(16) $$\begin{array}{c} x_{cl}^{Q}\cap x_{cl}^{R}=0,\;\;\;\;if\;\;Q\neq R \end{array}$$

(17) $$\begin{array}{c} p\left(x_{cl}^{Q}\right)=p_{0}\left(Q\right) \end{array}$$

(18) $$\begin{array}{c} \phi \left(X^{Q}\right)=p\left(X_{cl}^{Q}|x_{cl}^{Q}\right) \end{array}$$

The intuitive meaning of the above proposition can be summarized as follows. A set of incompatible observables, represented by noncommuting self-adjoint operators in Γ, is selected for measurement according to the probabilities $p_{0}\left(Q\right)$ defined in (14). This process of selection and measurement is modeled by classical events $x_{cl}^{Q}$ and $X_{cl}^{Q}$, respectively: $x_{cl}^{Q}$ corresponds to the event of setting up the measurement device for observable *Q*, while $X_{cl}^{Q}$ represents a specific measurement outcome of *Q*. Condition (15) ensures that an outcome can occur only if the corresponding measurement device has been properly set up. Condition (16) reflects the incompatibility of observables *Q* and *R* by requiring that the events $x_{cl}^{Q}$ and $x_{cl}^{R}$ are disjoint—that is, incompatible observables cannot be measured simultaneously. Condition (17) guarantees that the classical model reproduces the intended probabilities $p_{0}\left(Q\right)$ for the selection of measurements. Finally, condition (18) captures the central claim of the hypothesis: quantum probabilities can be expressed as classical conditional probabilities—specifically, as the probabilities of measurement outcomes conditioned on the setup of the appropriate measurement device.

Let us apply the Kolmogorovian Censorship Hypothesis to the EPR–Bohm scenario. We will do it in two different ways: first *without* and then *with* the common causes. In the first case, the set Γ contains four noncommuting self-adjoint operators:

(19) $$\begin{array}{c} \left\{\vec{\sigma }{\vec{a}}_{m}⊗\vec{\sigma }{\vec{b}}_{n},\;m,n=1,2\right\} \end{array}$$

each with four spectral projections:

(20) $$\begin{array}{c} \left\{A_{m}B_{n},\;A_{m}B_{n}^{⊥},\;A_{m}^{⊥}B_{n},\;A_{m}^{⊥}B_{n}^{⊥},\;m,n=1,2\right\} \end{array}$$

Let $p_{0}$ assign to each unit operator a probability $p_{mn}$ such that $\sum_{mn}p_{mn}=1$.

Now, the classical probability space (Ω,Σ,p) is generated by the measurement settings $a_{m}$, $b_{n}$ and measurement outcomes $A_{m},A_{m}^{⊥}$, $B_{n},B_{n}^{⊥}$ and their conjunctions (m,n=1,2). (For simplicity, we denote both the outcome event and the projection representing it in quantum mechanics by the same symbol, $A_{m},B_{n}$. However, the quantum state ϕ or the classical probability *p* will always indicate in which sense we use it.) Their relation can be characterized as follows:

(21) $$\begin{array}{ccc} A_{m}\wedge A_{m}^{⊥}=B_{n}\wedge B_{n}^{⊥} & = & \emptyset \;\;m,n=1,2 \end{array}$$

(22) $$\begin{array}{ccc} A_{m}\vee A_{m}^{⊥} & = & a_{m}\;\;m=1,2 \end{array}$$

(23) $$\begin{array}{ccc} B_{n}\vee B_{n}^{⊥} & = & b_{n}\;\;n=1,2 \end{array}$$

(24) $$\begin{array}{ccc} a_{1}\wedge a_{2}=b_{1}\wedge b_{2} & = & \emptyset \end{array}$$

(25) $$\begin{array}{ccc} a_{1}\vee a_{2}=b_{1}\vee b_{2} & = & \Omega \end{array}$$

(26) $$\begin{array}{ccc} p(a_{m}\wedge b_{n}) & = & p_{mn}\;\;m,n=1,2 \end{array}$$

(27) $$\begin{array}{ccc} \phi (A_{m}) & = & p(A_{m}|a_{m})\;\;m=1,2 \end{array}$$

(28) $$\begin{array}{ccc} \phi (A_{m}^{⊥}) & = & p(A_{m}^{⊥}|a_{m})\;\;m=1,2 \end{array}$$

(29) $$\begin{array}{ccc} \phi (B_{n}) & = & p(B_{n}|b_{n})\;\;n=1,2 \end{array}$$

(30) $$\begin{array}{ccc} \phi (B_{n}^{⊥}) & = & p(B_{n}^{⊥}|b_{n})\;\;n=1,2 \end{array}$$

(31) $$\begin{array}{ccc} \phi (A_{m}B_{n}) & = & p(A_{m}\wedge B_{n}|a_{m}\wedge b_{n})\;\;m,n=1,2 \end{array}$$

(32) $$\begin{array}{ccc} \phi (A_{m}^{⊥}B_{n}) & = & p(A_{m}^{⊥}\wedge B_{n}|a_{m}\wedge b_{n})\;\;m,n=1,2 \end{array}$$

(33) $$\begin{array}{ccc} \phi (A_{m}B_{n}^{⊥}) & = & p(A_{m}\wedge B_{n}^{⊥}|a_{m}\wedge b_{n})\;\;m,n=1,2 \end{array}$$

(34) $$\begin{array}{ccc} \phi (A_{m}^{⊥}B_{n}^{⊥}) & = & p(A_{m}^{⊥}\wedge B_{n}^{⊥}|a_{m}\wedge b_{n})\;\;m,n=1,2 \end{array}$$

As we see, the Kolmogorovian Censorship Hypothesis translates the EPR–Bohm scenario (9)–(11) into its well-known classical conditional form:

(35) $$\begin{array}{ccc} p(A_{m}|a_{m}) & = & \frac{1}{2} \end{array}$$

(36) $$\begin{array}{ccc} p(B_{n}|b_{n}) & = & \frac{1}{2} \end{array}$$

(37) $$\begin{array}{ccc} p(A_{m}\wedge B_{n}|a_{m}\wedge b_{n}) & = & \frac{1}{2}\sin^{2}\left(\frac{\theta_{mn}}{2}\right) \end{array}$$

resulting in conditional correlations

(38) $$\begin{array}{ccc} p(A_{m}\wedge B_{n}|a_{m}\wedge b_{n}) & \neq & p\left(A_{m}|a_{m}\right)\;p\left(B_{n}|b_{n}\right) \end{array}$$

for non-perpendicular directions.

Now, due to linearity of ϕ

(39) $$\begin{array}{ccc} \phi (A_{m}) & = & \phi \left(A_{m}B_{n}\right)+\phi \left(A_{m}B_{n}^{⊥}\right) \end{array}$$

(40) $$\begin{array}{ccc} \phi (B_{n}) & = & \phi \left(A_{m}B_{n}\right)+\phi \left(A_{m}^{⊥}B_{n}\right) \end{array}$$

and hence using (27)–(34) one obtains

(41) $$\begin{array}{ccc} p(A_{m}|a_{m}) & = & p\left(A_{m}\wedge B_{n}|a_{m}\wedge b_{n}\right)+p\left(A_{m}\wedge B_{n}^{⊥}|a_{m}\wedge b_{n}\right)=p\left(A_{m}|a_{m}\wedge b_{n}\right) \end{array}$$

(42) $$\begin{array}{ccc} p(B_{n}|b_{n}) & = & p\left(A_{m}\wedge B_{n}|a_{m}\wedge b_{n}\right)+p\left(A_{m}^{⊥}\wedge B_{n}|a_{m}\wedge b_{n}\right)=p\left(B_{n}|a_{m}\wedge b_{n}\right) \end{array}$$

which is just classical no-signaling.

In the second case, we apply the Kolmogorovian Censorship Hypothesis to the EPR–Bohm scenario taking into account the common causes ${\left\{C_{k}\right\}}_{k\in K}$. Assume that for each $C_{k}$, $\phi (C_{k})\neq 0$. What is the classical conditional translation of this common causal explanation?

First, observe that each $C_{k}$ introduces a new state on N by

(43) $$\begin{array}{c} \phi_{C_{k}}\left(X\right)=\frac{\phi (C_{k}XC_{k})}{\phi (C_{k})} \end{array}$$

Now, repeat the above procedure with each state $\phi_{C_{k}}$; that is, generate a classical probability space $\left(\Omega_{k},\Sigma_{k},p_{k}\right)$ such that (21)–(34) hold, *modulo* replacing ϕ with $\phi_{C_{k}}$; *p* with $p_{k}$; and $A_{m},B_{n},a_{m},n_{n}$ with $A_{mk},B_{nk},a_{mk},b_{nk}$. Note that due to (26) each $p_{k}$ will assign the same probability $p_{mn}$ to each measurement setting $a_{mk}\wedge b_{nk}$.

Since $\phi_{C_{k}}\left(C_{k}\right)=1$, the state $\phi_{C_{k}}$ can be interpreted as the conditional state, a state conditioned on the event $C_{k}$. To obtain the whole probability space, we need to ’glue together’ the |K| probability spaces (where |K| is the cardinality of the set *K*). Define the new sample space $\Omega^{\prime }$ as the disjoint union of sample spaces $\Omega_{k}$:

(44) $$\begin{array}{c} \Omega^{\prime }:=\underset{k\in K}{⋃}\;\left(\Omega_{k}\times \left\{k\right\}\right) \end{array}$$

and the events in $\Omega^{\prime }$ as follows:

(45) $$\begin{array}{ccc} A_{m} & := & \underset{k\in K}{⋃}\;\left(A_{mk}\times \left\{k\right\}\right) \end{array}$$

(46) $$\begin{array}{ccc} B_{n} & := & \underset{k\in K}{⋃}\;\left(B_{nk}\times \left\{k\right\}\right) \end{array}$$

(47) $$\begin{array}{ccc} a_{m} & := & \underset{k\in K}{⋃}\;\left(a_{mk}\times \left\{k\right\}\right) \end{array}$$

(48) $$\begin{array}{ccc} b_{n} & := & \underset{k\in K}{⋃}\;\left(b_{nk}\times \left\{k\right\}\right) \end{array}$$

and

(49) $$\begin{array}{ccc} C_{k} & := & \Omega_{k}\times \left\{k\right\} \end{array}$$

Define the probability $p^{\prime }$ as follows:

(50) $$\begin{array}{ccc} p^{\prime }\left(C_{k}\right) & := & \phi (C_{k}) \end{array}$$

(51) $$\begin{array}{ccc} p^{\prime }\left(a_{m}\wedge b_{n}|C_{k}\right) & := & p_{k}\left(a_{mk}\wedge b_{nk}\right)=p_{mn} \end{array}$$

(52) $$\begin{array}{ccc} p^{\prime }\left(A_{m}|a_{m}\wedge C_{k}\right) & := & p_{k}\left(A_{mk}|a_{mk}\right)=\phi_{C_{k}}\left(A_{m}\right) \end{array}$$

(53) $$\begin{array}{ccc} p^{\prime }\left(B_{n}|b_{n}\wedge C_{k}\right) & := & p_{k}\left(B_{nk}|b_{nk}\right)=\phi_{C_{k}}\left(B_{n}\right) \end{array}$$

(54) $$\begin{array}{ccc} p^{\prime }\left(A_{m}\wedge B_{n}|a_{m}\wedge b_{n}\wedge C_{k}\right) & := & p_{k}\left(A_{mk}\wedge B_{nk}|a_{mk}\wedge b_{nk}\right)=\phi_{C_{k}}\left(A_{m}B_{n}\right) \end{array}$$

Note that (51) expresses no-conspiracy and observe that (39) and (40) will hold also for each $\phi (C_{k})$ and hence (41) and (42) will hold for every $p_{k}$.

With this construction we arrive at a so-called *local, non-conspiratorial joint common causal explanation* of the conditional correlations (38), that is a partition $\left\{C_{k}\right\}$ of Ω such that for any $m,m^{\prime },n,n^{\prime }=1,2$ the following requirements hold:

(55) $$\begin{array}{ccc} p^{\prime }\left(A_{m}\wedge B_{n}|a_{m}\wedge b_{n}\wedge C_{k}\right)=p^{\prime }\left(A_{m}|a_{m}\wedge b_{n}\wedge C_{k}\right)\;p^{\prime }\left(B_{n}|a_{m}\wedge b_{n}\wedge C_{k}\right) & & \left(\mathrm{screening}\text{-}\mathrm{off}\right) \end{array}$$

(56) $$\begin{array}{ccc} p^{\prime }\left(A_{m}|a_{m}\wedge b_{n}\wedge C_{k}\right)=p^{\prime }\left(A_{m}|a_{m}\wedge b_{n^{\prime }}\wedge C_{k}\right) & & \left(\mathrm{locality}\right) \end{array}$$

(57) $$\begin{array}{ccc} p^{\prime }\left(B_{n}|a_{m}\wedge b_{n}\wedge C_{k}\right)=p^{\prime }\left(B_{n}|a_{m^{\prime }}\wedge b_{n}\wedge C_{k}\right) & & \left(\mathrm{locality}\right) \end{array}$$

(58) $$\begin{array}{ccc} p^{\prime }\left(a_{m}\wedge b_{n}\wedge C_{k}\right)=p^{\prime }\left(a_{m}\wedge b_{n}\right)\;p^{\prime }\left(C_{k}\right) & & \left(\mathrm{no}\text{-}\mathrm{conspiracy}\right) \end{array}$$

The rationale for conditions (55)–(58) is as follows. *Screening-off* (55) represents the application of the common cause principle to conditional correlations: although $A_{m}$ and $B_{n}$ are correlated when conditioned on $a_{m}$ and $b_{n}$, this correlation vanishes upon further conditioning on $C_{k}$. *Locality* in (56) and (57) expresses the natural expectation that a measurement outcome on one side should depend only on the local measurement setting and the value of the common cause, and not on the distant measurement choice. Lastly, *no-conspiracy* (58) requires that the common cause system be probabilistically independent of the measurement settings.

Just as in the quantum case, from (55)–(58) one can derive the Clauser–Horne inequality

(59) $$\begin{array}{c} -1⩽p^{\prime }\left(A_{1}\wedge B_{1}|a_{1}\wedge b_{1}\right)+p^{\prime }\left(A_{1}\wedge B_{2}|a_{1}\wedge b_{2}\right)+p^{\prime }\left(A_{2}\wedge B_{1}|a_{2}\wedge b_{1}\right)\\ -p^{\prime }\left(A_{2}\wedge B_{2}|a_{2}\wedge b_{2}\right)-p^{\prime }\left(A_{1}|a_{1}\wedge b_{1}\right)-p^{\prime }\left(B_{1}|a_{1}\wedge b_{1}\right)⩽0 \end{array}$$

(The proof is similar to that of (12); see Appendix B.) Note that in the last step of the derivation we used the following equations:

(60) $$\begin{array}{ccc} p^{\prime }\left(A_{m}|a_{m}\right) & = & \sum_{k}p^{\prime }\left(A_{m}|a_{m}\wedge C_{k}\right)\;p^{\prime }\left(C_{k}\right) \end{array}$$

(61) $$\begin{array}{ccc} p^{\prime }\left(B_{n}|b_{n}\right) & = & \sum_{k}p^{\prime }\left(B_{n}|b_{n}\wedge C_{k}\right)\;p^{\prime }\left(C_{k}\right) \end{array}$$

(62) $$\begin{array}{ccc} p^{\prime }\left(A_{m}\wedge B_{n}|a_{m}\wedge b_{n}\right) & = & \sum_{k}p^{\prime }\left(A_{m}\wedge B_{n}|a_{m}\wedge b_{n}\wedge C_{k}\right)\;p^{\prime }\left(C_{k}\right) \end{array}$$

which hold because of no-conspiracy (58).

## 4. Conclusions

Up to this point we did not assume that the common cause is commuting. For a noncommuting common cause the above classical probability space $\left(\Omega^{\prime },\Sigma^{\prime },p^{\prime }\right)$ can be constructed just as well as for a commuting common cause and the Clauser–Horne inequality (59) will follow. Where noncommutativity kicks in is how the classical probabilities *p* constructed *without* the common causes and the probability $p^{\prime }$ constructed *with* the common causes relate to one another. Namely, the equations

(63) $$\begin{array}{ccc} p(A_{m}|a_{m}) & = & p^{\prime }\left(A_{m}|a_{m}\right) \end{array}$$

(64) $$\begin{array}{ccc} p(B_{n}|b_{n}) & = & p^{\prime }\left(B_{n}|b_{n}\right) \end{array}$$

(65) $$\begin{array}{ccc} p(A_{m}\wedge B_{n}|a_{m}\wedge b_{n}) & = & p^{\prime }\left(A_{m}\wedge B_{n}|a_{m}\wedge b_{n}\right) \end{array}$$

will hold if and only if

(66) $$\begin{array}{c} \phi \left(X\right)=\sum_{k}\phi_{C_{k}}\left(X\right)\phi \left(C_{k}\right)\;\;X=A_{m},B_{n},A_{m}B_{n} \end{array}$$

hold. But for noncommuting common causes (66) cannot hold for all m,n=1,2; otherwise, the Clauser–Horne inequality (12) would follow. This means that for noncommuting common causes we will have two different Clauser–Horne inequalities depending on whether we plug in the right- or the left-hand side of (63)–(65) into (59), that is the probabilities obtained by applying the Kolmogorovian Censorship Hypothesis to the EPR–Bohm scenario without or with the common causes. Call these two inequalities *p*-Clauser–Horne inequalities and $p^{\prime }$-Clauser–Horne inequalities, respectively. Now, the difference between these two inequalities is the following. The $p^{\prime }$-Clauser–Horne inequality (59) can be derived and does hold for every common cause; while the *p*-Clauser–Horne inequality is violated but cannot be derived for noncommuting common causes. This means that the violation of the *p*-Clauser–Horne inequality composed of the original surface probabilities does not exclude a local, non-conspiratorial joint common causal explanation constructed from noncommuting common causes. And this is due to the violation of the law of total probability.

Obviously, this fact can also be expressed in a less triumphant tone: if the law of total probability is violated, the correlations which we would like to explain are not the same as the correlations which we arrive at using the common causes. We explain something other than what we wanted to explain. This is indeed bad news for noncommuting common causes. We address this problem in another paper [11]. This flaw, however, is not a consequence of the noncommuting common causes being conspiratorial. And that is all what we wanted to show in this paper.

## Data Availability

No new data were created or analyzed in this study. Data sharing is not applicable to this article.

## Appendix A

**Proof.** It is an elementary fact of arithmetic that for any $\alpha_{1},\alpha_{2},\beta_{1},\beta_{2}\in \left[0,\;1\right]$ the number

(A1) $$\begin{array}{c} \alpha_{1}\beta_{1}+\alpha_{1}\beta_{2}+\alpha_{2}\beta_{1}-\alpha_{2}\beta_{2}-\alpha_{1}-\beta_{1} \end{array}$$

lies in the interval [−1 ,0]. Now, substituting the expressions

(A2) $$\begin{array}{ccc} \alpha_{1} & := & \frac{\phi (C_{k}A_{1}C_{k})}{\phi (C_{k})} \end{array}$$

(A3) $$\begin{array}{ccc} \alpha_{2} & := & \frac{\phi (C_{k}A_{2}C_{k})}{\phi (C_{k})} \end{array}$$

(A4) $$\begin{array}{ccc} \beta_{1} & := & \frac{\phi (C_{k}B_{1}C_{k})}{\phi (C_{k})} \end{array}$$

(A5) $$\begin{array}{ccc} \beta_{2} & := & \frac{\phi (C_{k}B_{2}C_{k})}{\phi (C_{k})} \end{array}$$

into (A1) and using (8) we get

(A6) $$\begin{array}{c} -1⩽\frac{\phi (C_{k}A_{1}B_{1}C_{k})}{\phi (C_{k})}+\frac{\phi (C_{k}A_{1}B_{2}C_{k})}{\phi (C_{k})}+\frac{\phi (C_{k}A_{2}B_{1}C_{k})}{\phi (C_{k})}\\ -\frac{\phi (C_{k}A_{2}B_{2}C_{k})}{\phi (C_{k})}-\frac{\phi (C_{k}A_{1}C_{k})}{\phi (C_{k})}-\frac{\phi (C_{k}B_{1}C_{k})}{\phi (C_{k})}⩽0. \end{array}$$

Multiplying the above inequality by $\phi (C_{k})$ and summing up for the index *k* one obtains (12). □

## Appendix B

**Proof.** Consider the expressions

(A7) $$\begin{array}{ccc} \alpha_{1} & := & p^{\prime }\left(A_{1}|a_{1}\wedge b_{1}\wedge C_{k}\right) \end{array}$$

(A8) $$\begin{array}{ccc} \alpha_{2} & := & p^{\prime }\left(A_{2}|a_{2}\wedge b_{2}\wedge C_{k}\right) \end{array}$$

(A9) $$\begin{array}{ccc} \beta_{1} & := & p^{\prime }\left(B_{1}|a_{1}\wedge b_{1}\wedge C_{k}\right) \end{array}$$

(A10) $$\begin{array}{ccc} \beta_{2} & := & p^{\prime }\left(B_{2}|a_{2}\wedge b_{2}\wedge C_{k}\right) \end{array}$$

and plug them again into (A1). Using locality (56) and (57) one obtains

(A11) $$\begin{array}{c} -1⩽p^{\prime }\left(A_{1}|a_{1}\wedge b_{1}\wedge C_{k}\right)p^{\prime }\left(B_{1}|a_{1}\wedge b_{1}\wedge C_{k}\right)+p^{\prime }\left(A_{1}|a_{1}\wedge b_{2}\wedge C_{k}\right)p^{\prime }\left(B_{2}|a_{1}\wedge b_{2}\wedge C_{k}\right)\\ +p^{\prime }\left(A_{2}|a_{2}\wedge b_{1}\wedge C_{k}\right)p^{\prime }\left(B_{1}|a_{2}\wedge b_{1}\wedge C_{k}\right)-p^{\prime }\left(A_{2}|a_{2}\wedge b_{2}\wedge C_{k}\right)p^{\prime }\left(B_{2}|a_{2}\wedge b_{2}\wedge C_{k}\right)\\ -p^{\prime }\left(A_{1}|a_{1}\wedge b_{1}\wedge C_{k}\right)-p^{\prime }\left(B_{1}|a_{1}\wedge b_{1}\wedge C_{k}\right)⩽0 \end{array}$$

and using screening-off (55) one gets

(A12) $$\begin{array}{c} -1⩽p^{\prime }\left(A_{1}\wedge B_{1}|a_{1}\wedge b_{1}\wedge C_{k}\right)+p^{\prime }\left(A_{1}\wedge B_{2}|a_{1}\wedge b_{2}\wedge C_{k}\right)+p^{\prime }\left(A_{2}\wedge B_{1}|a_{2}\wedge b_{1}\wedge C_{k}\right)\\ -p^{\prime }\left(A_{2}\wedge B_{2}|a_{2}\wedge b_{2}\wedge C_{k}\right)-p^{\prime }\left(A_{1}|a_{1}\wedge b_{1}\wedge C_{k}\right)-p^{\prime }\left(B_{1}|a_{1}\wedge b_{1}\wedge C_{k}\right)⩽0 \end{array}$$

Multiplying the above inequality by $p^{\prime }\left(C_{k}\right)$, using no-conspiracy (58) and summing up for the index *k* one obtains (59). □
